# Effector Vδ1 γδ T cells mediate anti-tumor immunity and are reprogrammed by imatinib in human gastrointestinal stromal tumor

**DOI:** 10.21203/rs.3.rs-9557741/v1

**Published:** 2026-06-01

**Authors:** Shan Zeng, Jonathan H. Sussman, Montana Morris, Yuntian Fu, Juan Esteban Perez, Jiazhen Rong, Katherine Tardy, Hyunjee Kwak, Taylor Hartlein, Shujing Liu, Kevin Do, Ferdinando Rossi, Jennifer Q. Zhang, Xiaowei Xu, Ronald P. DeMatteo

**Affiliations:** 1 Department of Surgery, Perelman School of Medicine, University of Pennsylvania, Philadelphia, PA; 2 Graduate Group in Genomics and Computational Biology, Perelman School of Medicine, University of Pennsylvania, Philadelphia, PA; 3 Medical Scientist Training Program, Perelman School of Medicine, University of Pennsylvania, Philadelphia, PA; 4 Department of Statistics and Data Science, Perelman School of Medicine, University of Pennsylvania, Philadelphia, PA; 5 Department of Pathology, Perelman School of Medicine, University of Pennsylvania, Philadelphia, PA

**Keywords:** γδ T cells, Vδ1 T cells, Single-cell transcriptomics, T cell receptor (TCR) repertoire, Gastrointestinal stromal tumor, Tumor immunity

## Abstract

Gamma-delta (γδ) T cells are emerging effectors of anti-tumor immunity, but their relevance to gastrointestinal stromal tumor, the most common human sarcoma, is not defined. Here, we integrate single-cell transcriptomics, immunophenotypic and T cell receptor profiling, and functional assays of circulating and intratumoral γδ T cells across 68 human GIST specimens. Vδ1 cells predominated within tumors, and their abundance correlated with improved outcomes across 3 separate patient cohorts. γδ T cells from untreated tumors were cytotoxic *ex vivo*, but tumors that had become resistant to the tyrosine kinase inhibitor imatinib had lower Vδ1 composition, effector function, and altered clonality, and were enriched for apoptosis, IL-17 signaling, and checkpoint pathways. In a genetically engineered murine GIST model, PD-L1 blockade enhanced imatinib efficacy and restored tumor γδ T cell function. Thus, γδ T cells contribute to tumor immunity in GIST and are reprogrammed during imatinib resistance, making them an attractive immunotherapy target.

## Introduction

Gastrointestinal stromal tumor (GIST) is the most common human sarcoma and usually harbors either a *KIT* or *PDGFRA* mutation^1^. The tyrosine kinase inhibitor (TKI) imatinib mesylate improves survival but is rarely curative due to acquired drug resistance, which typically occurs within 2 years^1^. Although we found that imatinib initially increased intratumoral CD8^+^ T cells in a genetically engineered *Kit*^*V558del/+*^ mouse model of GIST^2,3^, in human GIST *CD4* and *CD8A* expression were not prognostic^4^ and conventional T cell checkpoint blockade was ineffective^5^; these findings prompted us to examine other immune effectors.

γδ T cells recognize a variety of antigens on tumor cells and unlike αβ T cells, exert cytotoxicity independently of HLA^6^. There are different subsets of human γδ T cells based on their γ and δ chains. Each TCRδ chain associates with one or more dominant TCRγ chains. The Vγ9Vδ2 subset predominates in human peripheral blood and can be readily expanded *in vivo* and *in vitro*. In contrast, human solid tissues are enriched for Vδ2-negative γδ T cells, primarily Vδ1 cells, which perform immune surveillance and possess cytotoxic potential^6^. A smaller Vδ3 subset, most often paired with Vγ2, has been described, although its functional role is unclear. Effects of γδ T cells in human solid tumors depend on tumor type and γδ T cell subset composition^7^. Anti-tumor effects are driven through interferon-γ (IFNγ) production and innate-like cytotoxicity; increased γδ T cell tumor infiltration has been associated with better clinical outcome in some malignancies^8,9^. However, in other contexts, tumor-infiltrating γδ T cells may acquire inhibitory phenotypes associated with impaired effector function and reduced IFNγ secretion^10^. IL17A-producing γδ T cells have variable effects on tumors, as they are anti-tumoral in colorectal cancer^11^ but mediate tumor resistance in breast cancer^12^. In murine models, single-cell RNA sequencing and γδ TCR profiling have demonstrated that γδ cell function is strongly influenced by TCR usage^13^. Individual γδ TCRs have an intrinsic ability to participate in both innate and adaptive immune features driven by CDR3δ length and composition^14^.

Here, we establish a γδ T cell atlas in human GIST by integrating single-cell transcriptomics, immunophenotyping, TCR profiling, and functional assays of circulating and tumor-infiltrating γδ T cells across 68 tumor samples. Our findings establish γδ T cells as a core component of GIST immunosurveillance and provide a framework for γδ T cell-directed immunotherapy in GIST.

## Results

### Single-cell transcriptomics reveal γδ T cell heterogeneity.

**Fig. 1:**
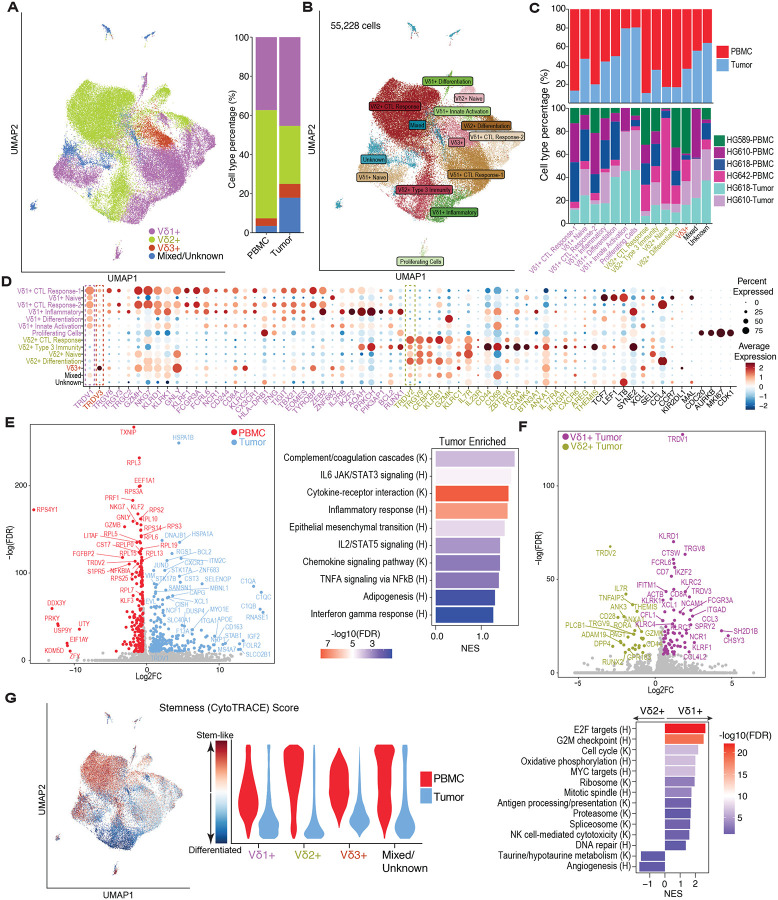
scRNA-seq analysis of γδ T cells in PBMCs and tumors from patients with GIST. γδ T cells were sorted from PBMCs and tumor specimens and processed for 10x Genomics scRNA-seq. **A) *left*,** UMAP of γδ T cells annotated by broad class (Vδ1, Vδ2, Vδ3) defined by canonical marker expression. **B)** UMAP of γδ T cells annotated by cluster (55,228 cells across 4 PBMC and 2 tumor samples). **C) *top*,** Bar plots showing the distribution of each cell cluster across PBMC and tumor samples and ***bottom,*** across the 6 samples. **D)** Dot plot of selected differentially expressed genes in each cluster. Genes relevant to Vδ1, Vδ2, and Vδ3 subsets are indicated by color. Dot size indicates the percentage of cells in the cluster expressing the gene, and color indicates Z-score-normalized average expression between clusters. **E)** Differentially expressed genes between PBMCs and tumor samples shown as ***left*** a volcano plot and ***right*** gene set enrichment (GSEA) analysis of differentially expressed pathways. Representative significant pathways (FDR<0.1) are shown. No pathways were significantly upregulated in PBMC samples. **F) *top,*** Volcano plot of differentially expressed genes between V*δ*1 and V*δ*2 cells within tumor samples. ***bottom,*** GSEA analysis of differentially expressed pathways between tumor V*δ*1 and V*δ*2 cells. Representative significant pathways (FDR<0.1) are shown. **E-F)** Hallmark (H) and KEGG (K) pathways are indicated in parentheses. FDR, false discovery rate. NES, normalized enrichment score. **G)** CytoTRACE stemness score across γδ T cells shown as ***left*** UMAP and ***right*** violin plots separated by V*δ*1, V*δ*2, and V*δ*3 subsets in PBMCs and tumor samples.

To dissect the role of γδ T cells in human GIST, we sorted CD45^+^CD3^+^TCRγδ^+^ cells from untreated human GISTs and matched peripheral blood mononuclear cells (PBMCs) to profile them using single-cell RNA sequencing (scRNA-seq) (**Fig. S1, S2A-B**). After quality control, we obtained 55,228 cells from 4 blood and 2 tumor samples. We initially defined Vδ1, Vδ2, and Vδ3 T cell clusters based on canonical marker (*TRDV1*, *TRDV2*, and *TRDV3*) expression (Fig. 1A). Vδ2 cells were more prevalent in PBMCs, while Vδ1 and Vδ3 subsets were more frequent in tumors, consistent with reports that Vδ2-negative cells predominate within solid tumors^15,16^. Flow cytometry in 13 patients revealed that the percentages of V*δ*1 T cells correlated between PBMCs and tumors (**Fig. S3**).

Unbiased clustering of our integrated tumor/PBMC γδ T cell atlas demonstrated 7 clusters for V*δ*1, 4 for V*δ*2, and 1 for Vδ3 based on differential gene expression (Fig. 1B–C, **S2C-D, Table S1**). The Vδ1-Cytotoxic T lymphocyte (CTL) cluster highly expressed classic markers for effector function (Fig. 1D), such as cytotoxicity (*GZMH*, *NKG7*, *KLRD1*, *KLRK1*, *GNLY, KLRF1*) and other effector and exhaustion markers (*FCGR3A*, *FCRL6, NCR1* (NKp46)*, CD244, CD8A, KLRC2, IFNG, and TIGIT*). There was also high expression of *TBX21*, *ZEB2, and EOMES*, the master transcriptional regulators of conventional T cell effector function. In contrast, the Vδ1-naive cluster possessed high expression of innate markers (*TCF7*, *LEF1*, *LTB*, *XCL1*, *CCR7*, *MAL*) as well as the transcription factor *ZNF683* encoding *PRDM1* (BLIMP1) that regulates T cell differentiation and lineage. The V*δ*1-Inflammatory cluster had the highest expression of *KLRK1* (NKG2D), *IL2RB*, and *IKZF2* (Helios) and increased expression of genes involved in T cell-mediated inflammation and activation (*AOAH*, *PRKCH*, *ZEB2*, *PIK3AP1*, *BCL2*, *RUNX1*). The V*δ*3 subset most closely resembled the V*δ*1-CTL cluster and had the highest expression of killer-cell immunoglobulin-like receptors (KIRs) and *GNLY*, implying cytotoxicity based on HLA class I expression.

Of the 4 V*δ*2 T cell clusters, V*δ*2-CTL predominated and was enriched for hallmark V*δ*2 markers (*CEBPD*, *KLRB1*, *GZMK*, *KLRC1, IL7R*, and *CD23R*)^17^. Compared to V*δ*1-CTL, V*δ*2-CTL had lower expression of cytotoxic molecules, exhaustion markers, and *IFNG*. Another V*δ*2 cluster highly expressed *CD44*, *CD69*, *ZBTB16* (encoding *PLZF*), *RORA*, *RUNX1, CAMK4,* a calmodulin-dependent protein kinase (CaMK), and *BTN3A1*, an essential membrane protein for TCR–dependent activation of human V*γ*9V*δ*2 T cells^18^. This V*δ*2 cluster was also highly enriched for other unique markers (e.g., *ANXA1*, *IL17RA*, *IL23R, IFNGR1*, *CXCR6*, *AREG*, *THEMIS*) and was annotated as V*δ*2-type 3 immunity, based on a prior report^19^. Smaller populations of V*δ*2 T cells (V*δ*2-Naïve and V*δ*2-Differentiation) shared some common gene markers with V*δ*2-CTL, but showed higher expression of *SELL* and *CCL4*, respectively.

Tumor γ*δ* T cells had unique gene expression programs compared to PBMC γ*δ* T cells (Fig. 1E, **left**), including heat shock proteins (*HSPA1B* and *HSPA1A*), stress response genes (*DNAJB1*), the integrin *ITGA1*, and other differentiation-related genes (e.g., *ZNF683, XCL1*, *DUSP4*, and *CXCR3*). Gene set enrichment analysis (GSEA) revealed multiple signaling pathways in tumor-infiltrating versus circulating γδ T cells, such as cytokine-receptor interactions, inflammatory response, IL2/STAT5 signaling, and IFNγ (Fig. 1E, **right**). Compared to tumor Vδ2 cells, tumor Vδ1 cells upregulated most V*δ*1 signature genes (e.g. *TRDV1*, *TRDV3*, *TRGV8*, *KLRD1*, *FCGR3A, KLRC2, KLRF1, IKZF2, FCRL6, XCL1, NCR1, ITGAD*) and other markers of cytotoxicity and exhaustion. Similarly, tumor V*δ*2 cells showed increased expression of V*δ*2 signature genes (*TRDV2, TRGV9, IL7R, THEMIS, ANXA1, RORA,* and *RUNX2*; Fig. 1F, **top**). GSEA demonstrated that tumor Vδ1 cells were enriched for cell cycle pathways, oxidative phosphorylation, antigen processing, and NK cell-mediated cytotoxicity (Fig. 1F, **bottom**), the latter two of which were also enriched in circulating V*δ*1 cells (**Fig. S2E**). Lastly, using CytoTRACE to infer relative differentiation states among γδ T cell populations^20^, tumor-infiltrating γδ T cells were more differentiated than circulating γδ T cells (Fig. 1G). Taken together, transcriptomes of purified PBMC and tumor γδ T cells of GIST patients indicated considerable heterogeneity and broad functional plasticity.

### Regulatory network analysis identifies Vδ1 γδ T cell transcriptional programs.

**Fig. 2:**
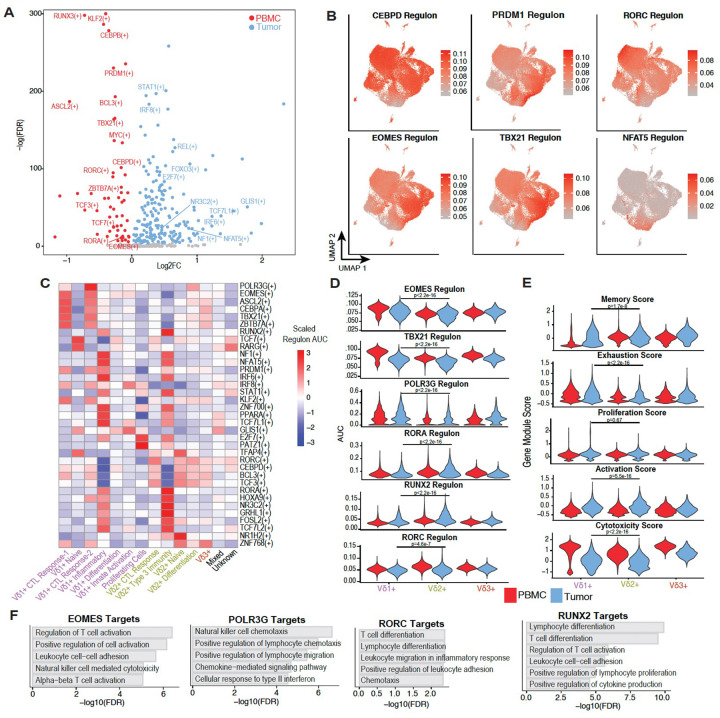
Regulon activity and potential gene-regulatory networks based on SCENIC of PBMC and tumor γδ T cells. **A)** Differential transcriptional regulons as inferred by SCENIC between PBMCs and tumor γδ T cells. **B)** UMAP visualization of γδ T cells colored by regulon activity scores calculated using AUCell across the dataset. **C)** Heatmap of selected regulon activity across γδ T cells subsets. Regulon activity is Z-score normalized by row. **D)** Violin plots of selected regulon activities across PBMC and tumor γδ T cells stratified by broad class (Vδ1, Vδ2, Vδ3). Significance between tumor V*δ*1 and tumor V*δ*2 cells was assessed using the Wilcoxon rank-sum test. **E)** Gene module scores of broad γδ T cell classes within PBMCs and tumors as in **D**. **F)** Enrichment analysis using Gene Ontology pathways was conducted on the predicted target genes of each regulon. Selected significant pathways are shown.

To investigate the transcription factors driving γδ T subsets, we used single-cell regulatory network interference and clustering (SCENIC) to identify potential regulatory mechanisms^21^. Regulons mediating lymphocyte trafficking (e.g., RUNX3, KLF2, PRDM1, CEBPD) were highly activated in circulating γδ T cells across all clusters (Fig. 2A–B). Predicted CEBPD target genes included both V*δ*1 and V*δ*2 signature genes (e.g., *ZEB2, ZBTB16, ITGA1, THEMIS, RORA, IL7R*), which mediate T cell differentiation and activation (Fig. 2C). In addition, regulons of EOMES, TBX21 (Tbet), and RORC, which regulate conventional T cell (T-conv) activation, were also higher in PBMC γδ T cells (Fig. 2A). On further analysis, RORC was mainly detectable in V*δ*2 (Fig. 2B–C), but not Vδ1 cells. Conversely, EOMES and TBX21 were primarily in the V*δ*1 subset, especially in the Vδ1-CTL cluster (Fig. 2C), suggesting they functionally resembled CD8^+^ *αβ* T cells. Regulons linked to inflammation or T cell exhaustion induced by stress antigens (e.g., STAT1, NFAT5, IRF8) were enriched across all tumor-infiltrating γδ T cells, suggesting presence of IFNγ signaling, inflammation, and exhausted T cell differenation^22–24^.

Tumor V*δ*1 and V*δ*2 T cells had distinct regulon activity (Fig. 2D, **S4**). EOMES, TBX21, and POLR3G were enriched in V*δ*1 cells, particularly in the V*δ*1-CTL cluster (Fig. 2C). NFAT5 and IRF6 were specifically increased in the V*δ*1-Inflammatory cluster. In contrast, RORC was more activated in the tumor V*δ*2 subset. RORA and RUNX2 primarily regulated the V*δ*2-Type 3 immunity phenotype (Fig. 2C). To identify potential functional differences mediated by these regulons, we computed immune module scores based on selected gene markers for memory, exhaustion, proliferation, activation, and cytotoxicity (**Table S2**)^25–27^. Tumor V*δ*1 cells had higher exhaustion, activation, and cytotoxicity scores, whereas tumor V*δ*2 cells had higher memory scores (Fig. 2E). Lastly, enrichment analysis using Gene Ontology was conducted based on predicted target genes of each regulon (Fig. 2F). Consistent with their transcriptional profiling, targets of EOMES and POLR3G were enriched for T cell activation, cytotoxicity, and lymphocyte chemotaxis. Meanwhile, RORC and RUNX2 were associated with pathways in T cell differentiation, activation, and leukocyte adhesion. In summary, we defined diverse functional phenotypes in γδ T cells and inferred their regulatory mechanisms.

### γδ TCR analysis uncovers clonotype diversity and TKI alteration.

**Fig. 3:**
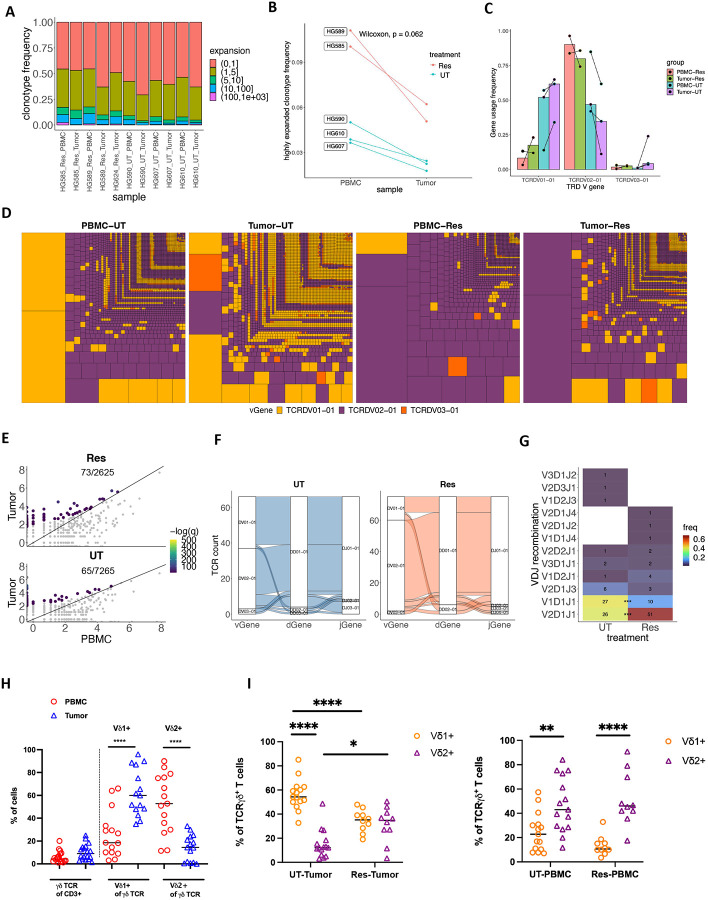
Compositional and clonal analysis of γδ T cells in PBMCs and tumors. γδ T cells were sorted from human PBMC and GISTs. gDNA was processed for TCRαδ CDR3 next-generation sequencing. **A**) Clonotype frequency in all specimens (n=11). **B**) Frequency of highly expanded (>10) clonotypes in 5 matched PBMC and tumors. **C**) Frequency of TCRDV gene segment usage in matched untreated (UT) and resistant (Res) specimens. **D**) Tree plots illustrating the composition of TCR repertoires in matched specimens. Each square represents a clone, with its area proportional to the clone size and its color indicating the V gene usage. **E**) Scatter plot of cell count distribution of a clone in PBMC versus tumor. Each point is a clone and the x,y axes denote the log transformed cell count in PBMC and tumor. Clones which have significantly high expansion are highlighted with FDR controlled q value. **F**) The alluvium plot for the VDJ combination of clones which have significantly high expansion in untreated and resistant tumors. **G**) The frequency change between the untreated and resistant patient groups for each VDJ recombination in clones which are highly expanded in tumor. VDJ recombinations with significant change are highlighted with “***”. **H**) Frequency of Vδ1 and Vδ2 γδ T cells determined by flow cytometry in PBMCs and tumors. **I**) ***left,*** Frequency of Vδ1 and Vδ2 γδ T cells in tumors or ***right,*** PBMCs based on treatment status in untreated (UT; n=15), and resistant (Res; n=10) specimens. Medians indicated. For all panels, * *p*<0.05, ** *p*<0.01, *** *p*<0.001; **** *p*<0.0001.

To examine the γδ TCR repertoire, we performed next-generation sequencing of genomic DNA for TCR delta chains in sorted γδ T cells from 5 pair-matched PBMC and tumor samples (3 untreated and 2 with acquired resistance to imatinib) and 1 additional resistant tumor. Most (>75%) of the PBMC and tumor γδ TCRs were minimally expanded with fewer than 5 clones (Fig. 3A). Patients with resistant tumors had a higher frequency of highly expanded clones (clone size >10), particularly in the blood (Fig. 3B).

In 4 of 5 patients, TCRDV1 usage was higher in tumors than paired PBMCs (Fig. 3C), consistent with our scRNA-seq data. Notably, TCRDV1 usage was lower in the 2 tumors that developed resistance to imatinib compared to the 3 untreated tumors, while TCRDV2 gene usage was higher in the 2 resistant tumors. The tree plot summarizes TCRDV usage. TCRDV1 usage was higher in tumors than PBMCs in the untreated samples, both overall and in highly expanded clones. Furthermore, it was higher in untreated versus resistant patients in the tumor compartment by both metrics (Fig. 3D). We next performed differential expansion analysis for each clone between paired PBMC and tumor samples (Fig. 3E). In resistant tumors, there was a higher frequency of highly expanded clones compared to untreated tumors (2.78 vs. 0.89%, *p*=3.91×10^−11^). The recombinations V1D1J1 and V2D1J1 predominated in both groups, suggesting that the primary variation lies in V gene usage (Fig. 3F). In the untreated group, V1D1J1 and V2D1J1 appeared at a similar frequency, whereas in the resistant group, V2D1J1 was enriched while V1D1J1 was reduced (Fig. 3G). To confirm our findings, we performed flow cytometry for Vδ1 and Vδ2 in human PBMCs and tumors in 29 patients. Consistent with our scRNA-seq and TCR sequencing in sorted γδ T cells, the Vδ1 subset dominated in the tumor, whereas the V*δ*2 subset was more abundant in paired PBMCs (Fig. 3H). Furthermore, TKI-resistant GISTs had lower tumor V*δ*1 and higher V*δ*2 composition than untreated tumors (Fig. 3I, **left**), even though the relative proportion of the 2 subsets did not vary with treatment status in PBMCs (Fig. 3I, **right**). Overall, resistant tumors had an altered γδ TCR repertoire.

### Tumor Vδ1 T cells are cytotoxic and prognostic of clinical outcome.

**Fig. 4:**
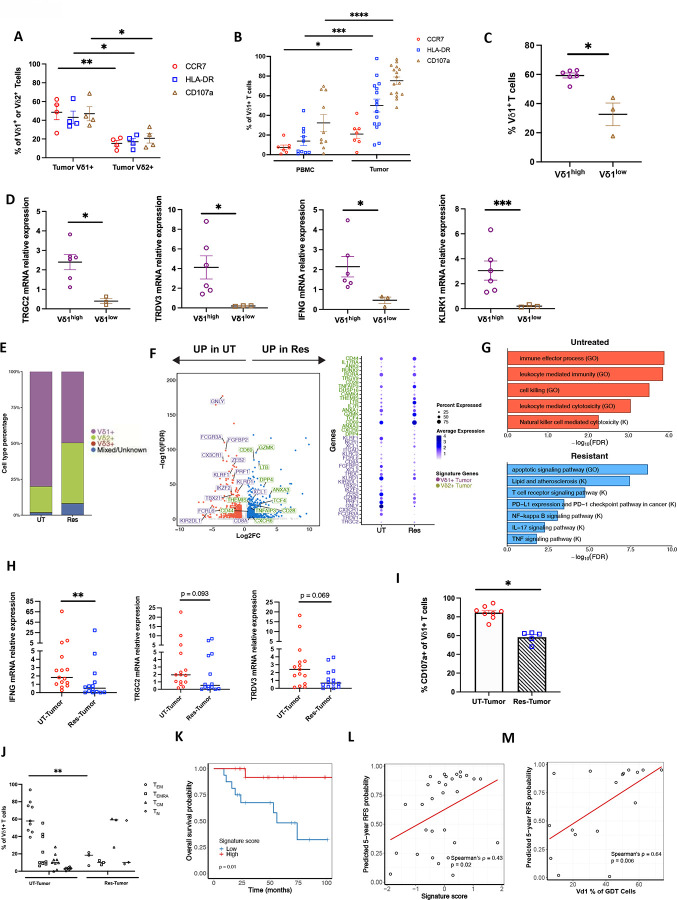
Tumor-infiltrating V*δ*1 T cells exhibit enhanced cytotoxicity and are prognostic of improved clinical outcomes. **A**) Comparison of selected marker expression by flow cytometry between tumor-infiltrating V*δ*1 and V*δ*2 T cells. Data represent mean±SEM. **B**) Summary of selected markers in PBMC-derived (n=10) and tumor-derived (n=8–16) V*δ*1 T cells. Data represent mean±SEM. **C**) Tumor γδ T cells were sorted and grouped by relatively high V*δ*1 (mean 59.2%, n=6) or low V*δ*1 frequency (mean 32.6%, n=3) using a cut-off of 50% V*δ*1 frequency. **D**) qPCR showing mRNA expression of *TRGC2*, *TRDV3*, *IFNG* and *KLRK1* in sorted tumor γδ T cells with high or low Vδ1 proportions. Data represent mean±SEM. **E**) Composition of γδ T cell subsets (n = 6,970 cells) in scRNA-seq data from untreated (n=3) and resistant (n=2) GISTs based on sorted γδ T cell reference (Fig. 1A). **F**) ***left,*** Volcano plot of differentially expressed genes between γδ T cells in untreated (n=3) and resistant (n=2) GISTs based on scRNA-seq data. Key V*δ*1 and Vδ2 marker genes (Fig. 1F) are highlighted. ***right,*** Corresponding Dot plot of V*δ*1 and V*δ*2 marker genes. **G**) Over representation analysis using Gene Ontology (GO), KEGG (K), and Reactome (R) gene sets in the top differentially expressed genes between untreated (***top***) and resistant γδ T cells (***bottom***). Representative significant pathways are shown (FDR <0.05). **H**) qPCR analysis showing relative mRNA expression of *TRGC2*, *TRDV3* and *IFNG* in untreated (n=15) and resistant (n=14) GISTs. Medians indicated. **I**) Flow cytometry analysis of CD107a expression on tumor Vδ1 γδ T cells from untreated (UT; n=8) and resistant GISTs (Res; n=5). Data represent mean ±SEM. **J)** Summary data of effector status of tumor Vδ1 T cells from untreated (UT; n=9) and imatinib-resistant tumors based on flow cytometry (Res; n=3). Medians indicated. T_EM_ - effector memory T cells, T_EMRA_, - terminally differentiated effector memory T cells, T_CM_ - central memory T cells, T_N_ – naïve T cells. **K**) Kaplan-Meier overall survival analysis of a published Japanese cohort of 32 patients with untreated primary GISTs, stratified by median expression score of γδ T cell signature genes (*TRGC2*, *TRDC*, *TRDV3, and TRGV2*). **L**) Scatterplot of predicted 5-year recurrence-free survival (RFS) based on median expression score of γδ T cell signature genes in our published cohort of bulk RNA-seq in 30 untreated primary GISTs. **M**) Scatterplot of predicted 5-year RFS in 17 patients with untreated primary GIST based on Vδ1 percentage of γδ T cells determined by flow cytometry. For all panels, *p<0.05; **p<0.01; ***p<0.001, **** *p*<0.0001.

To validate our scRNA-seq atlas, which showed effector and exhaustion markers in the V*δ*1 subset, we performed flow cytometry on 10 untreated GISTs. Tumor-infiltrating V*δ*1 T cells expressed CD8A, FCGR3A (encoding CD16), PDCD1 (PD1), TIGIT, and EOMES (**Fig. S5A**), as identified in Fig. 1D. In addition, expression of CCR7, HLA-DR, and the cytotoxic degranulation marker CD107a was higher in tumor-infiltrating V*δ*1 cells compared to V*δ*2 cells (Fig. 4A, **S5B**) and PBMC Vδ1 cells (Fig. 4B), indicating enhanced tumor trafficking, activation, and cytotoxic potential. To assess the functional relevance of tumor-infiltrating V*δ*1 cells *ex vivo*, we sorted γδ T cells from 9 additional human GISTs (7 untreated and 2 imatinib-resistant). Six tumors had a high proportion of V*δ*1 cells among γδ T cells (mean 59.2%), while 3 had V*δ*1 lower percentages (mean 32.6%) (Fig. 4C). Tumors with a high V*δ*1 proportion had increased expression of *TRGC2*, *TRDV3*, *IFNG* and *KLRK1* (encoding NKG2D) (Fig. 4D), thereby validating our scRNA-seq findings shown in Figure 1D.

The TCR data of sorted γδ T cells suggested that resistant GISTs had altered clonotype diversity and subset composition. Therefore, we performed additional scRNA-seq analysis on 3 untreated and 2 resistant GISTs, without sorting γδ T cells. CD3^+^ T cells were identified and clustered across all samples (**Fig. S6A**). Two clusters were annotated as γδ T cells based on canonical gene expression (**Fig. S6B**). Using reference mapping from our γδ T cell scRNA-seq atlas (Fig. 1A), we identified a substantial shift in γδ T cell subset composition, from Vδ1 dominance in untreated tumors to a more balanced Vδ2 proportion in resistant tumors (Fig. 4E). Differential gene expression analysis revealed enrichment of tumor-specific Vδ1 signature genes in untreated tumor γδ T cells and Vδ2 genes in resistant γδ T cells (Fig. 1F, 4F, **left)**. Notably, higher expression of cytotoxic effector genes, including *GNLY*, *PRF1*, *KLRF1*, *KLRD1*, and *FCGR3A*, was observed in untreated tumors, indicating enhanced cytotoxic activity (Fig. 4F, **right**). Over-representation enrichment analysis (ORA) confirmed that untreated tumor γδ T cells were significantly enriched for immune effector processes, leukocyte-mediated immunity, cell killing, and NK cell-mediated cytotoxicity. In contrast, γδ T cells in resistant tumors had increased apoptosis, T cell receptor signaling, and lipid metabolism, as well as PD-1/PD-L1 checkpoint, NF-kappa B, and IL-17 signaling pathways (Fig. 4G).

To further validate these findings, we analyzed RNA from 29 human GISTs (15 untreated and 14 resistant tumors) by quantitative real-time PCR (qPCR). Expression of *TRGC2* and *TRDV3*, reflecting the abundance of V*δ*1 and V*δ*3 cells, respectively, trended lower in imatinib-resistant tumors than in untreated GISTs (Fig. 4H), corroborating our flow cytometry and scRNA-seq data showing reduced V*δ*1 cells in resistant GISTs, with concordant IFNG reduction. CD107a expression was decreased in tumor-infiltrating V*δ*1 cells from resistant GISTs (Fig. 4I), indicating reduced effector function. V*δ*1 T cells in untreated tumors predominantly displayed an effector phenotype, with high frequencies of T cell effector memory (T_EM_) populations, in contrast to resistant tumors (Fig. 4J).

To determine the clinical relevance of tumor V*δ*1 T cells in GIST, we analyzed 3 independent cohorts of untreated patients. In a bulk tumor RNA microarray of 32 untreated primary human GISTs from Japan^28^, we applied a published γδ T cell signature (*TRGC2*, *TRDC*, *TRDV3, and TRGV2*)^29^ and stratified high versus low signature expression based on the median. High signature expression was associated with improved overall survival (Fig. 4K). Next, we evaluated this γδ T cell signature in our published cohort of 30 untreated primary GISTs^30^ and found that it correlated with predicted recurrence-free survival (RFS)^31^ (Fig. 4L). Finally, we quantified Vδ1 T cell abundance by flow cytometry in 17 untreated primary GIST tumors and again found a correlation to predicted RFS (Fig. 4M). Thus, γδ T cells, and V*δ*1 T cells in particular, are favorably prognostic in untreated human GIST.

### γδ T cells are cytotoxic in patient-derived tumor models.

**Fig. 5:**
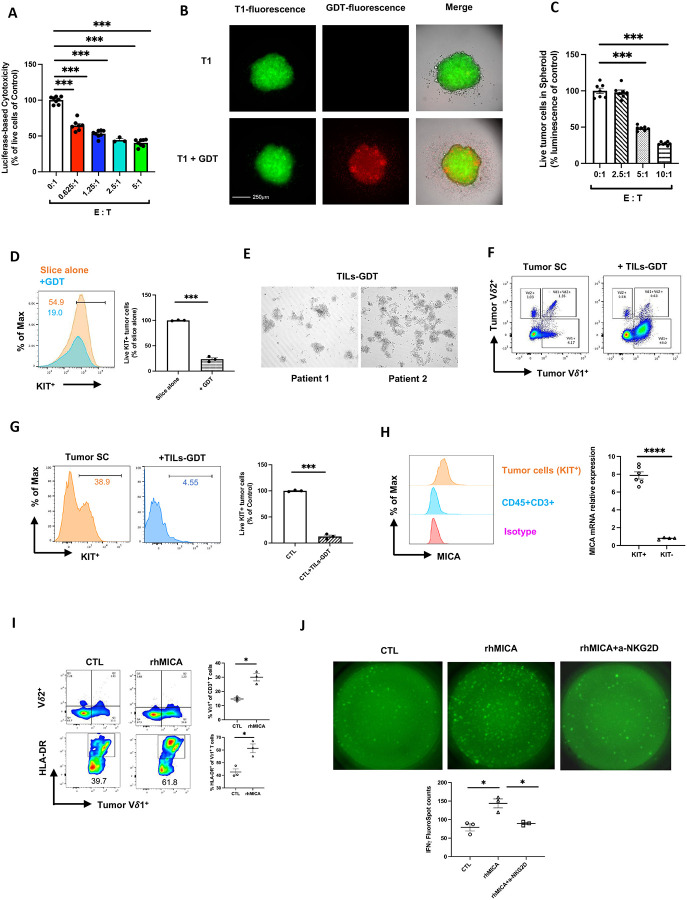
Expanded circulating and tumor-infiltrating γδ T cells exhibit cytotoxicity against GIST. **A**). Expanded γδ T cells from healthy donors were co-cultured with luciferase-expressing GIST-T1 cells at the indicated effector-target (E:T) ratios. After 24h, cytotoxicity was assessed by measuring luciferase activity. **B**) Representative images of a tumor spheroid assay. GIST-T1 cell spheroids were co-cultured with expanded γδ T cells, E:T ratio of 2.5:1, for 24h. Tumor cells expressed luciferase (green), and γδ T cells were labeled with CellTrace (red). **C**) Cytotoxicity in spheroids after co-culture with γδ T cells for 72h at the indicated E:T ratios as measured by luciferase assay. **D**) ***left*,** Histograms of KIT expression on tumor cells and ***right,*** quantification of KIT^+^ tumor cell killing in human GIST tissue slices co-cultured with expanded γδ T cells at an E:T ratio of 5:1 for 24h, assessed by flow cytometry after tissue slice dissociation. **E**) Representative pictures showing expansion of tumor-derived γδ T cells on day 6. Tumor-infiltrating lymphocytes (TILs) from untreated GISTs were isolated using CD45 microbeads and expanded with anti-γδ TCR antibody supplemented with IL-2 (200IU/ml). **F**) Representative flow plots showing robust expansion of Vδ1 cells at 24h following co-culture of expanded tumor-derived γδ T cells with tumor single-cell suspensions (tumor SC). **G**) Cytotoxicity against autologous KIT^+^ tumor cells was evaluated by co-culturing expanded tumor-derived γδ T cells as shown in **F**. ***left,*** Flow cytometry histograms of KIT expression on tumor cells and ***right***, quantification of KIT+ tumor cell killing. **H**) ***left*,** Flow cytometry histograms of MICA expression on tumor cells (KIT^+^) and T cells (CD45^+^CD3^+^) and ***right,*** qPCR showing relative *MICA* mRNA expression in KIT^+^ tumor cells and KIT^−^ cells from GIST patients. **I**) Tumor-infiltrating lymphocytes (TILs) were treated with recombinant MICA (rhMICA, 10 μg/ml) and IL-2 for 72h and analyzed by flow cytometry, demonstrating an increased percentage of Vδ1 γδ T cells, and greater frequency of HLA-DR among the same. **J**) Sorted tumor γδ T cells from untreated GISTs were co-cultured with rhMICA or anti-NKG2D antibody for 24h, and IFNγ production was measured by ELISplot. Data represent mean±SEM, For all panels, *p<0.05; **p<0.01; ***p<0.001, **** *p*<0.0001.

To assess whether γδ T cells kill GIST tumors, we first expanded PBMC-derived γδ T cells from healthy donors (n=4) with an anti-*γδ* TCR antibody and IL-2 (**Fig. S7A**)^32^. Healthy donors with a high frequency of Vδ1 cells after 8 days of expansion (**Fig. S7B**) were used for *in vitro* assays (n=2). Expanded γδ T cells demonstrated dose-dependent cytotoxicity against luciferase-expressing human GIST T1 cells (Fig. 5A). Fluorescently labeled γδ T cells migrated into GIST spheroids and accomplished tumor cell killing, albeit at a higher E:T ratio (Fig. 5B–C). To evaluate γδ T cell function in a more physiologic setting, we utilized an *ex vivo* human GIST tumor slice model. Primary tumor slices from 3 untreated patients were co-cultured with expanded γδ T cells from healthy donors. After 24h, tissue slices were dissociated into single-cell suspensions and viable KIT^+^ tumor cells were quantified by flow cytometry. Expanded γδ T cells infiltrated tumor slices (**Fig. S7C**) and reduced KIT^+^ tumor cells (Fig. 5D).

Although tumor-infiltrating γδ T cells are thought to possess limited anti-tumor activity in certain cancers due to their exhausted phenotypes^6^, our scRNA-seq data revealed enrichment of a NK cell-mediated cytotoxicity pathway in tumor Vδ1 cells. Therefore, we expanded tumor-infiltrating lymphocytes (TILs) from 2 untreated GIST patients for 6 days using an anti-*γδ* TCR antibody and IL-2, and cultured them with matched tumor single-cell suspensions for 24h (Fig. 5E–F). Co-culture reduced the number of viable KIT^+^ tumor cells (Fig. 5G). Given that tumor-associated ligands can activate γδ T cells, we examined the role of MICA, an NKG2D ligand expressed by KIT^+^ tumor cells in human GIST (Fig. 5H). Plate-bound recombinant MICA plus IL-2 expanded V*δ*1 cells within TILs and induced higher expression of HLA-DR (Fig. 5I). MICA stimulation also induced IFNγ production, which was attenuated by NKG2D blockade (Fig. 5J). Thus, both circulating and tumor-infiltrating γδ T cells, particularly the V*δ*1 subset, retain cytotoxic effector function following expansion and activation.

### Initial imatinib response alters tumor γδ T cell composition and function in mice.

**Fig. 6:**
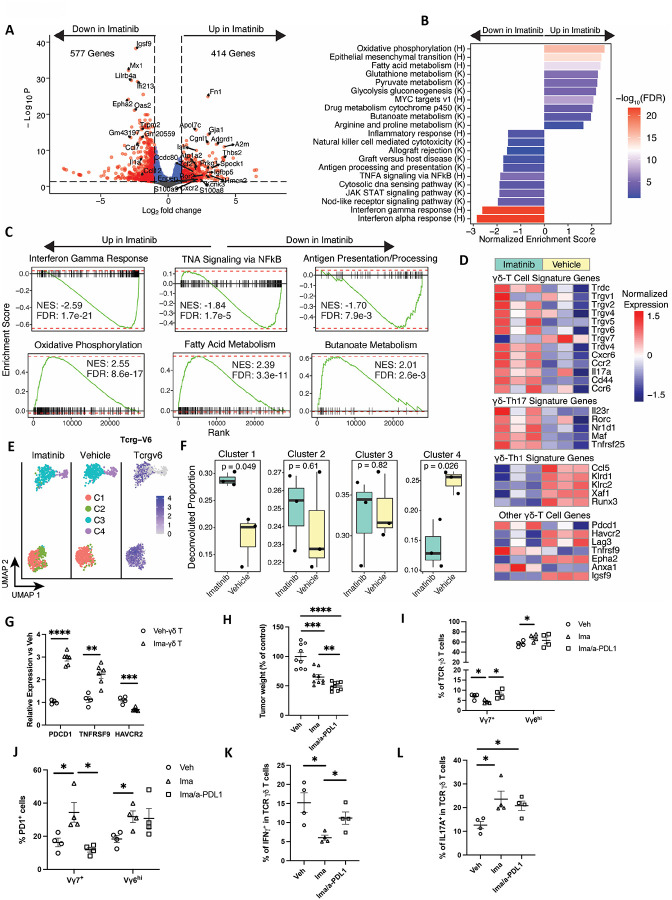
Imatinib drives functional and compositional reprogramming of tumor γδ T cells *in vivo*. γδ T cells were sorted from *Kit*^*V558del/+*^ mice treated with vehicle or imatinib for 1 week and processed for bulk RNA-seq. **A)** Volcano plot of differentially expressed genes in tumor γδ T cells based on treatment (n=3/group). Significant genes defined with p-adjusted < 0.05 and log_2_FC > 1. **B)** Gene set enrichment analysis (GSEA) of Hallmark (H) and KEGG (K) pathways in tumor γδ T cells based on treatment. **C)** Selected enrichment plots from **B**. NES, normalized enrichment score; FDR, false discovery rate. **D)** Heatmap of selected tumor γδ T cell signature genes across samples. Color represents Z-score normalized expression for each gene. **E)** UMAP of previously published scRNA-seq data of murine γδ T cells (1,641) cells, separated by treatment and colored by *Tcrgv6* gene expression. **F)** Deconvolution of bulk RNA-seq data with the single-cell reference in **E** using CIBERSORTx. Significance was assessed using a Welch’s 2-sided t-test. **G**) qPCR of expression of selected genes from sorted γδ T cells. n=4–6/group**. H)** Tumor weight after *Kit*^*V558del/+*^ mice were treated for 1 week with vehicle, imatinib, or imatinib plus anti-PDL1. n=9/group. **I)** The frequency of Vγ7 and Vγ6 high subsets among tumor γδ T cells from *Kit*^*V558del/+*^ mice treated in **H** as assessed by flow cytometry. **J)** The frequency of PD1^+^ cells in Vγ7 and Vγ6 high subsets among tumor γδ T cells in each group. **K-L)** The frequency of IFNγ^+^ cells (**K**) and IL17A^+^ cells (**L**) among tumor γδ T cells from *Kit*^*V558del/+*^ mice treated in **H**. Data represent mean±SEM. n=4/group. For all panels, *p<0.05, **p<0.01, ***p<0.001, ****p<0.0001.

Our human studies utilized untreated tumors and tumors that acquired resistance to imatinib. To investigate the early effect of imatinib on tumor γδ T cells, we administered imatinib for 1 week to *Kit*^*V558del/+*^ mice^3^, which develop an intestinal GIST. Bulk RNA-seq of sorted tumor γδ T cells showed that imatinib upregulated 414 genes and downregulated 577 (Fig. 6A). GSEA revealed increased oxidative phosphorylation, fatty acid metabolism, and glycolysis/gluconeogenesis pathways. Murine IL17A-secreting γδ T cells rely on oxidative lipid metabolism^33^, and previously we found that imatinib expanded mouse IL17A-producing γδ T cells^4^. Here, we further identified that imatinib reduced γδ T cell IFNγ response, antigen presentation, and NK cell-mediated cytotoxicity (Fig. 6B–C). IFNγ–producing murine γδ T cells, particularly the Vγ7/Vγ1 subset, display antitumor activity in various models, whereas IL-17A–producing γδ T cells, mainly the long-lived Vγ6 subsets, have variable pro-tumoral function^13^. In our model, by bulk gene expression and our published murine scRNA-seq data^4^, Vγ6 cells (cluster 1) predominated and were expanded by imatinib, while the Vγ7/Vγ1 subset (cluster 4) was reduced (Fig. 6D–F, **S8**). Accordingly, imatinib upregulated γδ-Th17 signature genes and reduced γδ-Th1 related and cytotoxicity genes (e.g., *Gzmb, Klrd1, Klrc2*), respectively. Bulk RNA-seq also showed that imatinib increased expression of *Pdcd1* (PD1) and *Tnfrsf9* (4–1BB ligand receptor), which were validated by qPCR (Fig. 6G).

Since checkpoint inhibition of PD-1 and PD-L1 can augment γδ T cell cytotoxicity in other solid tumors^16,34^, we hypothesized that targeting the PD1/PDL1 pathway would affect γδ T cell immunity in GIST. The combination of imatinib and an anti-PD-L1 blocking antibody further reduced tumor weight as expected^35^, but also restored the proportion of Vγ7^+^ cells, downregulated their PD1 expression, and increased γδ T cell IFNγ while maintaining IL-17 production (Fig. 6H–L). Taken together, these data suggest that checkpoint inhibition increased the anti-tumor efficacy of γδ T cells during imatinib response.

## Discussion

By integrating single-cell transcriptomic, TCR repertoire profiling, and functional analyses, we established a comprehensive framework for understanding tumor and circulating γδ T cell heterogeneity, differentiation, clonotype diversity, and function in GIST, revealing previously unappreciated mechanisms linking oncogenic signaling, innate-like cytotoxic immunity, and therapeutic response. Vδ1 cells were more prevalent in human tumor samples than in matched blood, regulated by EOMES and TBX21, and enriched for a T_EM_ phenotype. V*δ*1 cells expanded *in vitro* from healthy donor PBMCs killed tumor cells in 3 different patient-derived models, including freshly procured GIST tumor slices. TILs from GIST specimens expanded with an anti-TCR antibody were also cytotoxic against autologous tumor cells. The baseline γδ T frequency in PBMCs influence *ex vivo* expansion capacity^36^. In contrast, V*δ*2 cells were driven by RORC, predominated in tumors that had acquired resistance to imatinib, and expressed fewer effector molecules. Analysis of 3 independent patient cohorts demonstrated the prognostic value of total, as well as Vδ1-specific, γδ T cell abundance in untreated GISTs, as observed in other tumors.^29^ Notably, including Vδ1- and Vδ3-specific genes (*TRGC2* and *TRDV3*, respectively) improved upon the prognostication of *TRDC* alone, which we used previously^4^.

The TCR repertoire of sorted γδ T cells provides insight into the mechanisms underlying γδ T cell activation in GIST. Untreated GIST tumors displayed broad Vδ1 TCR diversity with relatively few highly expanded clones, implying limited engagement of a few dominant immunogenic tumor antigens. Known γδ TCR ligands, including Ephrin type-A receptor 2 (EphA2) and HLA/MHC class I chain-related proteins A and B (MICA/B), can trigger γδ T cell differentiation and expansion within tumors^37,38^. Indeed, MICA expression by human GIST tumor cells expanded the Vδ1 subset more efficiently than the Vδ2 subset and induced IFNγ production and HLA-DR upregulation. In contrast, the Vδ2 subset predominantly recognizes phosphoantigens (e.g., zoledronate) via the BTN2A1-BTN3A1 complex for expansion^39^. Admittedly, our TCR analysis focused on the δ chain, potentially underestimating clonotypic complexity. Nevertheless, these observations support the model that GIST contains multiple γδ immune circuits, with Vδ1 cells preferentially linked to tumor stress recognition and direct cytotoxic effector function.

Imatinib therapy remodeled the γδ T cell compartment in GISTs. While the Vδ1 subset was prevalent in untreated human GISTs, tumors that had acquired resistance to imatinib exhibited reduced Vδ1 representation, diminished T_EM_ differentiation, increased apoptosis, reduced cytotoxicity, altered cellular metabolism, and increased IL-17 signaling. These changes reinforce that while imatinib directly targets KIT signaling in tumor cells, the GIST tumor microenvironment is also remodeled, as we have shown previously with alterations in intratumoral T cells, tumor-associated macrophages, and dendritic cells^2,40,41^.

Our murine *Kit*^*V558del/+*^ model allowed us to investigate γδ T cells during the early (i.e., 1 week) tumor response to imatinib. Previously, we showed that γδ T cells in GIST contribute to tumor control through interleukin-17 (IL-17) production, as antibody-mediated depletion of either IL-17 or γδ T cells accelerated tumor growth in otherwise untreated mice^4^. The role of IL-17 in human GIST remains uncertain. Here, we found that the early response to imatinib in *Kit*^*V558del/+*^ mice upregulated tumor γδ T cell fatty acid metabolism and oxidative phosphorylation, as detected by bulk RNA-seq analysis of sorted γδ T cells. Furthermore, imatinib decreased tumor γδ T cell IFNγ in addition to increasing IL-17 production. Combining PD-L1 blockade with imatinib increased tumor cytotoxicity, tumor Vγ7 cells, and tumor γδ T cell IFNγ production. These findings are consistent with Vδ1 cells responding favorably to checkpoint inhibition in melanoma^16^ and Merkel cell carcinoma^34^. Human GIST tumor samples during early response to imatinib are not clinically available. Moreover, technical challenges precluded the isolation and analysis of sufficient γδ T cells after chronic therapy with imatinib, which is typically administered for at least 6 months prior to surgery to reduce tumor size.

Our study establishes Vδ1 γδ T cells as critical mediators of anti-tumor immunity in GIST and highlights γδ T cells as an actionable immunotherapeutic axis in GIST and other solid tumors that are refractory to conventional αβ T cell-directed immunotherapy. Emerging clinical efforts to harness γδ T cells, including CAR-engineered Vδ1 therapies currently under investigation^42,43^, further highlight the translational relevance of these immune cells.

## Methods

### Human GIST samples.

Tumor specimens and PBMCs were obtained from patients with GIST undergoing surgical resection under an Institutional Review Board-approved protocol (additional supporting data files). All patients provided written informed consent prior to surgery, and studies were conducted in accordance with the Declaration of Helsinki. Patient information was de-identified and encrypted in compliance with the Health Insurance Portability and Accountability Act regulations (HIPAA). Tumor tissues were sectioned and processed into single-cell suspensions. PBMCs were isolated from whole blood using CPT tubes and density gradient centrifugation.

### Cell isolation, sorting, and flow cytometry.

Single-cell suspensions were prepared from PBMCs, human GISTs, and murine GISTs derived from *Kit*^*V558del/+*^ mice. In brief, tumor tissues were minced and digested with a mixture of 2.5 mg/ml collagenase IV and 50 μg/ml DNase I at 37°C for 30 minutes. They were processed into single-cell suspensions as previously described 35. Flow cytometry was performed using an LSRFortessa (BD Biosciences). Prior to antibody staining, cells were incubated with an anti-CD16/32 antibody to block Fc receptors. The following fluorochrome-conjugated antibodies were used for murine cells: CD45 (30-F11), CD3 (17A2), CD4 (RM4–5), CD8 (53–6.7), NK1.1 (PK136), TCR *γδ* (GL3), IL17A (TC11–18H10.1), PD-1 (29F.1A12), IFN-γ (XMG1.2), TCR Vγ1 (2.11), Vγ2 (UC3–10A6), Vγ4 (49.2), Vγ6 (1C10–1F7), Vγ7 (F2.67). For human samples, the following antibodies were used: CD45 (2D1), CD3 (SK7), CD8 (RPA-TB), TCR γ*δ* (B1), TCR V*δ*1 (TS8.2), TCR V*δ*2 (B6), TCR Vγ9 (B3), TIGIT (TgMab-2), CD45RA (HI100), PD-1 (EH12.2H7), CD69 (FN50), IFN-γ (4S.B3), CCR7 (G043H7), HLA-DR (G46–6), CD27 (L128), CD107a (H4A3), NKG2D (1D11), MICA/B (6D4). Viable cells were identified using LIVE/DEAD Fixable Aqua (Themo Fisher Scientific). Transcription factor staining was performed with the Foxp3/Transcription Factor Staining Buffer Set (eBioscience), and intracellular cytokine staining was conducted using the Cytofix/Cytoperm Kit (BD Biosciences). For cell sorting, fluorescent-labeled antibodies were used to stain single-cell suspensions, and sorting was performed on a FACSAria III (BD Biosciences). Human γδ T cells were defined as CD45^+^CD3^+^TCRγδ^+^, and murine γδ T cells as CD45^+^CD3^+^NK1.1^−^TCRγδ^+^. Flow cytometry data were analyzed using FlowJo software (version 10.8.2; BD Biosciences).

### Human γδ T cell expansion and in vitro assays.

PBMCs were isolated from the peripheral blood of healthy donors or GIST patients. Tumor infiltrating lymphocytes (TILs) were isolated from tumor single-cell suspensions using CD45 microbeads (Miltenyi Biotec). For γδ T cell expansion, PBMCs or TILs (2x10^6^ cells per well) were seeded in 24-well plates pre-coated with anti-γδ TCR antibody (0.5 μg/ml; Immunotech) and cultured in RPMI1640 medium supplemented with IL-2 (200U/ml, PeproTech) for 6 to 10 days.

For *in vitro* cytotoxicity assays, luciferase-expressing GIST T1 cells (1x10^4^ per well) were seeded as target cells in 96-well flat-bottom plates. Expanded γδ T cells were added as effector cells at the indicated effector-to-target (E:T) ratio and co-cultured for 24h. Cells were then lysed, and cytotoxicity was quantified using the Luciferase Assay System (E1501, Promega) on a GloMax Discover Microplate Reader, according to manufacturer’s instructions. For three-dimensional (3D) spheroid assays, 96-well plates were pre-coated with 50 *μ*l of 1.0% agarose (Invitrogen). Luciferase-expressing GIST-T1 cells (5x10^3^ per well) were seeded and allowed to form spheroids for 24h. CellTrace-labeled γδ T cells (CellMask deep red, Invitrogen) were then added at the indicated E:T ratios. γδ T cell infiltration was analyzed by fluorescence microscopy at 24h and cytotoxicity was measured at 72h using the luciferase assay.

For GIST tissue slice co-culture assays, 6 mm tumor cores were collected in the operating room and maintained on ice. Specimens were embedded in low melting point agarose and sectioned into 300-μm slices using a vibrating microtome as described previously^44^. Tissue slices were placed into 0.4 μm Millicell inserts (Milipore) in 24-well plates and cultured in RPMI1640 medium supplemented with IL-2. One slice was dissociated to determine the number of target cells. CellTrace-labeled γδ T cells were added to the top of tissue slices at an E:T ratio 5:1. After 24h of co-culture, slices were washed of free cells with PBS and dissociated into single-cell suspensions with collagenase and DNase I for flow cytometry analysis. To assess cytotoxicity of tumor-derived γδ T cells against autologous KIT^+^ tumor cells, TILs were isolated and expanded for 6 days with an anti-γδ TCR antibody in the presence of IL2. Expanded TILs were then co-cultured with autologous tumor single-cell suspensions at an E:T ratio of 5:1 for 24h *in vitro*. Live KIT^+^ tumor cells were quantified by flow cytometry and normalized to tumor single-cell suspensions cultured alone as the control.

For human MICA stimulation, TILs were cultured with recombinant MICA (rhMICA; 10 *μ*g/ml; R&D Systems) or mouse IgG1 isotype control (50 ng/ml; BioLegend) for 72h and analyzed by flow cytometry. To examine the role of NKG2D signaling, sorted γδ T cells (predominantly Vδ1) from untreated GISTs were cultured in 96-well plates with rhMICA in the presence or absence of an anti-NKG2D blocking antibody (10 *μ*g/ml; clone 1D11; BioLegend) added prior to plating. IL-2 was included in the culture medium. After 24h, IFNγ production was measured by ELISpot (ImmunoSpot) following the manufacturer’s instructions.

### RNA isolation and real-time PCR.

Total RNA was extracted from human GIST specimens and murine *Kit*^*V558del/+*^ tumors using the RNeasy Mini Kit (Qiagen). Reverse transcription was performed with TaqMan reagents (Applied Biosystems). TaqMan gene expression assays (Applied Biosystems) included the following probes: mouse *Pdcd1* (Mm01285676_m1), *Havcr1* (Mm01294183_m1) and *Tnfrsf9* (Mm00441899_m1); and human *Trgc2* (Hs00827007_m1), *Trdv3* (Hs01669899_m1), *Ifng* (Hs009899291_m1), *Mica* (Hs00792195_m1), and *Klrk1* (Hs00183683_m1). qPCR was performed using a QuantStudio real-time PCR system (Applied Biosystems). Relative gene expression was calculated using the 2^−ΔΔCT^ method, as described in the manufacturer's instructions, and results were expressed as fold increase for murine samples or as median values for human samples relative to the indicated controls.

### Processing and quality control filtering of human γδ T cell scRNA-seq data.

Human γδT cells sorted from PBMCs and tumors were immediately processed for scRNA-seq using the 10x Genomics platform at the Center for Applied Genomics of the Children’s Hospital of Philadelphia. Read count matrices from scRNA-seq data were generated from raw FASTQ files using CellRanger v.7.1.0, with alignment to the GRCh38–2020-A human transcriptome reference. The resulting count matrices were processed and analyzed using Seurat v5^45^. Quality control filtering was applied to each cell, using filters of 100 < nFeatureRNA < 10000 mitochondrial read percentage < 10%. Doublets were then called using DoubletFinder (v2.0.4) using SCTransform normalization, an expected doublet rate of the total cell number / 125,000, and other parameters as default. Then, predicted doublets were removed and additional filters of nFeature_RNA > 300 and nCount_RNA > 1000 were applied.

### Sample integration, clustering, and cell type annotation of human γδ T cell scRNA-seq data.

Analysis of scRNA-seq data was conducted using Seurat v5^45^ and R v4.4.0. Samples were normalized individually using the SCTransform function with regression of the mitochondrial read percentage, and a PCA was computed. Then, samples were integrated using the IntegrateLayers function with RPCA integration and default parameters. A UMAP was constructed using the first 30 dimensions of the integrated reduction. Cells were clustered using the FindNeighbors function with the first 30 dimensions of the integrated reduction followed by the FindClusters function. The clustering resolution was chosen empirically. Cell cycle phase was predicted using the CellCycleScoring function after conversion of the default human gene lists to mouse orthologues. Reference mapping was performed with the FindTransferAnchors and Mapquery functions. Differential gene expression was conducted using the FindMarkers function with the default Wilcoxon rank-sum test on log-normalized counts, and clusters were manually annotated based on their differentially expressed genes. For construction of volcano plots, cells were downsampled to a maximum of 1,500 per condition to avoid p-values of zero. For pathway analysis, the log2FC across all genes was used as input to a pre-ranked gene set enrichment analysis (GSEA) using the fgsea package. Pathways were sourced from the Molecular Signatures Database (MsigDB) Hallmark gene sets^27^ and the KEGG database^46^. Overrepresentation enrichment analysis was performed on the top genes with predefined filtering parameters from select gene set collections using the gost function from the gprofiler2 package (minimum expressing cells = 20%, |log-fc| >1, FDR < 0.05). Differentiation trajectory was inferred using CytoTRACE (v0.3.3) with default parameters using the log-normalized counts.

### SCENIC transcriptional regulatory network (TRN) analysis.

The Single-Cell Regulatory Network Inference and Clustering (SCENIC) algorithm^47^ was used to identify transcription factors regulating γδ T cell states. It was run using the pySCENIC implementation as previously described^48^ on the scRNA-seq data, after conversion of the Seurat object to loom format. The GRNBoost2 algorithm was used for GRN inference. To predict transcription factor regulons, we used the human v9 cisTarget motif collection and the hg38_refseq-r80 databases with the 500bpUp100Dw and TSS+/−10kb search spaces. All relevant databases were obtained from: https://resources.aertslab.org/cistarget/. The pySCENIC implementation of AUCell was used to score the activity of regulons for each cell. The SCopeLoomR package (https://github.com/aertslab/SCopeLoomR) was then used to extract the regulons and AUCell matrix from the resulting loom file, and the final AUC matrix was added to the initial Seurat object for visualization and analysis. Differential regulons were identified using the FindAllMarkers function using the Wilcoxon rank-sum test. Gene sets enriched within regulon target genes were analyzed using the enrichGO function of clusterProfiler with the Biological Process Gene Ontology pathways.

### TCR Sequencing and analysis of TCRαδ repertoires.

Human γδ T cells were sorted from PBMCs (n=5) and tumor samples (n=6), and genomic DNA (gDNA) was extracted. Next-generation DNA sequencing of the CDR3 regions of human TCRα and TCRδ chains was performed using the ImmunoSEQ^®^ Assay (Adaptive Biotechnologies). gDNA was amplified by a multiplex PCR method, followed by high-throughput sequencing. Sequence output was filtered on in-frame CDR3s and normalized across all samples. All analyses were carried out using CDR3 amino sequences in the TCR*δ* chain to track clonal expansion within an individual. Briefly, to identify the TCR*δ* repertoires, data were pre-processed based on the productive rearranged TCRδ for each sample and TCRs with TCR*α* expression were first filtered out. Then, the count of TCR was quantified on the amino acid level for each sample. To measure clonotype frequency of TCR*δ* repertoires, we classified TCR into 5 expansion groups, using 1, 5, 10, 100 as boundaries according to the total count of TCRδ sequence in each sample. The frequency of each expansion group was calculated by the count of unique TCRs in the group divide by the total number of unique TCRs within the same sample. The frequency of the highly expanded group (clone size > 10) was calculated in the same way and compared between PBMC and tumor sample for each patient by the Wilcoxon rank-sum test. We applied Fisher’s exact test to assess clonal expansion between paired PBMC and tumor samples for each individual clone. To compare the number of clones significantly expanded in the tumor relative to PBMC between untreated and resistant patients, we also used Fisher’s exact test. Additionally, differences in the number of clones within each VDJ recombination group between the 2 patient groups were evaluated using the same test.

### Mice and treatments.

*Kit*^*V558del/+*^ mice (8–10 weeks old) on a B6 background (Jackson Laboratory) were maintained in a pathogen-free animal facility. Mice were age- and sex-matched and randomized for each experiment. Animal experiments were approved by the Institutional Animal Care and Use Committee at the University of Pennsylvania (IACUC). Imatinib mesylate (Novartis) was dissolved in the drinking water at 600 mg/L; isotype (rat IgG2b; BioXcell) and anti-PDL1 blocking antibody (10F.9G2; BioXcell) were administered intraperitoneally at a dose of 200 μg per mouse for 1 week as previously described^35^.

### Bulk RNA-seq analysis of murine γδ T cells.

To isolate γδ T cells from *Kit*^*V558del/+*^ mice, CD45^+^CD3^+^NK1.1^−^TCRγδ^+^ T cells were sorted from vehicle and imatinib treated mice using a FACSAria III (BD Biosciences). Total RNA was extracted, and next-generation RNA sequencing was performed on the Illumina HiSeq 2500 platform at the High-Throughput Sequencing Core of the Children’s Hospital of Philadelphia. Sequencing reads were aligned to the mouse genome (mm10) using STAR aligner with default parameters^49^. Expression was quantified using featureCounts^50^. Differentially expressed genes (DEGs) were identified using the DESeq2 package v1.46.0 and fold changes were shrunk with apeglm. The relevant differential expression coefficient statistic was used as input for Gene Set Enrichment Analysis (GSEA) using the fgsea^51^ package. Pathways were sourced from the Molecular Signatures Database (MsigDB)^52^. For a comparison to our published murine scRNA-seq data, we utilized CIBERSORTx^53^ using the scRNA-seq data as a reference with S-mode batch correction.

### Statistical analysis.

Statistical analyses for the scRNA-seq and TCRδ repertoires datasets are described above. Predicted 5-year recurrency free survival was calculated based on patient characteristics utilizing a published nomogram^31^. Overall survival analysis was performed using Kaplan-Meier method with the R package “survival.” Significance was assessed with Logrank test, and hazard ratio with a Cox proportional hazard model. Signature expression scores were calculated using a robust Z-scoring framework on normalized expression data from previously published datasets^28,30^. Each gene was median-centered and scaled by a robust spread estimate combining the standard deviation and median absolute deviation (MAD) across samples. Standardized values were then averaged across signature genes to yield a per-sample score. Correlations between continuous variables were evaluated using Spearman’s rank correlation coefficient. Differences between 2 experimental groups were analyzed using an unpaired, 2-tailed Welch’s *t*-test or Mann-Whitney U test. All *in vitro* and *ex vivo* experiments were performed using at least three biological samples. Statistics analyses were carried out using Prism 9.0 (Graph Pad Software) and R software (R Foundation for Statistical Computing). Data are shown as mean ± SEM or median, as specified in the Fig. legends. A *p* value < 0.05 was considered statistically significant.

## Supplementary Material

Supplementary Files

This is a list of supplementary files associated with this preprint. Click to download.

- MSSupplFigsFinal.pdf

## Data Availability

scRNA-seq data from sorted human γδ T cells and GIST tumors have been deposited to NCBI GEO under accession number GSE301351. Bulk RNA-seq data from sorted murine γδ T cells are available under accession number GSE300283. TCRαδsequencing data from sorted human γδ T cells are accessible via the ImmunoSEQ platform under the project name *UPenn_00131343_R-01*. Murine scRNA-seq used in this study were previously deposited in the Sequencing Read Archive (SRA) under accession number PRJNA859907. All remaining data supporting the findings of this study are available within the article and its supplementary materials or from the corresponding author upon reasonable request.

## Code availability.

Source code will be made available upon publication, and any code can be made available to the reviewers upon request.

## References

[1] Blay J.Y., Kang Y.K., Nishida T. & von Mehren M. Gastrointestinal stromal tumours. Nat Rev Dis Primers 7, 22 (2021). 33737510 10.1038/s41572-021-00254-5

[2] Balachandran V.P. Imatinib potentiates antitumor T cell responses in gastrointestinal stromal tumor through the inhibition of Ido. Nat Med 17, 1094–100 (2011). 21873989 10.1038/nm.2438 PMC3278279

[3] Sommer G. Gastrointestinal stromal tumors in a mouse model by targeted mutation of the Kit receptor tyrosine kinase. Proc Natl Acad Sci U S A 100, 6706–11 (2003). 12754375 10.1073/pnas.1037763100 PMC164511

[4] Etherington M.S. Tyrosine Kinase Inhibition Activates Intratumoral gammadelta T Cells in Gastrointestinal Stromal Tumor. Cancer Immunol Res 12, 107–119 (2024). 37922405 10.1158/2326-6066.CIR-23-0061 PMC10842124

[5] Singh A.S. A Randomized Phase II Study of Nivolumab Monotherapy or Nivolumab Combined with Ipilimumab in Patients with Advanced Gastrointestinal Stromal Tumors. Clin Cancer Res 28, 84–94 (2022). 34407970 10.1158/1078-0432.CCR-21-0878

[6] Hayday A., Dechanet-Merville J., Rossjohn J. & Silva-Santos B. Cancer immunotherapy by gammadelta T cells. Science 386, eabq7248 (2024). 10.1126/science.abq7248 PMC7616870 39361750

[7] Silva-Santos B., Mensurado S. & Coffelt S.B. gammadelta T cells: pleiotropic immune effectors with therapeutic potential in cancer. Nat Rev Cancer 19, 392–404 (2019). 31209264 10.1038/s41568-019-0153-5 PMC7614706

[8] Wu Y. A local human Vdelta1 T cell population is associated with survival in nonsmall-cell lung cancer. Nat Cancer 3, 696–709 (2022). 35637401 10.1038/s43018-022-00376-z PMC9236901

[9] Gentles A.J. The prognostic landscape of genes and infiltrating immune cells across human cancers. Nat Med 21, 938–945 (2015). 26193342 10.1038/nm.3909 PMC4852857

[10] Stary V. Dysfunctional tumor-infiltrating Vdelta1 + T lymphocytes in microsatellite-stable colorectal cancer. Nat Commun 15, 6949 (2024). 39138181 10.1038/s41467-024-51025-1 PMC11322529

[11] Wu P. gammadeltaT17 cells promote the accumulation and expansion of myeloid-derived suppressor cells in human colorectal cancer. Immunity 40, 785–800 (2014). 24816404 10.1016/j.immuni.2014.03.013 PMC4716654

[12] Petroni G. IL-17A-secreting gammadelta T cells promote resistance to CDK4/CDK6 inhibitors in HR(+)HER2(-) breast cancer via CX3CR1(+) macrophages. Nat Cancer 6, 1656–1675 (2025). 40624238 10.1038/s43018-025-01007-z

[13] Reis B.S. TCR-Vgammadelta usage distinguishes protumor from antitumor intestinal gammadelta T cell subsets. Science 377, 276–284 (2022). 35857588 10.1126/science.abj8695 PMC9326786

[14] Melandri D. The gammadeltaTCR combines innate immunity with adaptive immunity by utilizing spatially distinct regions for agonist selection and antigen responsiveness. Nat Immunol 19, 1352–1365 (2018). 30420626 10.1038/s41590-018-0253-5 PMC6874498

[15] Rancan C. Exhausted intratumoral Vdelta2(-) gammadelta T cells in human kidney cancer retain effector function. Nat Immunol 24, 612–624 (2023). 36928415 10.1038/s41590-023-01448-7 PMC10063448

[16] Davies D. PD-1 defines a distinct, functional, tissue-adapted state in Vdelta1(+) T cells with implications for cancer immunotherapy. Nat Cancer 5, 420–432 (2024). 38172341 10.1038/s43018-023-00690-0 PMC10965442

[17] Pizzolato G. Single-cell RNA sequencing unveils the shared and the distinct cytotoxic hallmarks of human TCRVdelta1 and TCRVdelta2 gammadelta T lymphocytes. Proc Natl Acad Sci U S A 116, 11906–11915 (2019). 31118283 10.1073/pnas.1818488116 PMC6576116

[18] Payne K.K. BTN3A1 governs antitumor responses by coordinating alphabeta and gammadelta T cells. Science 369, 942–949 (2020). 32820120 10.1126/science.aay2767 PMC7646930

[19] Tan L. A fetal wave of human type 3 effector gammadelta cells with restricted TCR diversity persists into adulthood. Sci Immunol 6(2021). 10.1126/sciimmunol.abf0125 33893173

[20] Gulati G.S. Single-cell transcriptional diversity is a hallmark of developmental potential. Science 367, 405–411 (2020). 31974247 10.1126/science.aax0249 PMC7694873

[21] Bravo Gonzalez-Blas C. SCENIC+: single-cell multiomic inference of enhancers and gene regulatory networks. Nat Methods 20, 1355–1367 (2023). 37443338 10.1038/s41592-023-01938-4 PMC10482700

[22] McMurray J.L. Transcriptional profiling of human Vdelta1 T cells reveals a pathogen-driven adaptive differentiation program. Cell Rep 39, 110858 (2022). 10.1016/j.celrep.2022.110858 PMC9533230 35613583

[23] Tille L. Activation of the transcription factor NFAT5 in the tumor microenvironment enforces CD8(+) T cell exhaustion. Nat Immunol 24, 1645–1653 (2023). 37709986 10.1038/s41590-023-01614-x

[24] Li R. Analysis of the three-dimensional genome of exhausted CD8(+) T cells reveals a critical role of IRF8 in their differentiation and functions in cancer. Nat Immunol 26, 2280–2295 (2025). 41254222 10.1038/s41590-025-02330-4

[25] Kurd N.S. Early precursors and molecular determinants of tissue-resident memory CD8(+) T lymphocytes revealed by single-cell RNA sequencing. Sci Immunol 5(2020). 10.1126/sciimmunol.aaz6894 PMC7341730 32414833

[26] Kumar B.V. Human Tissue-Resident Memory T Cells Are Defined by Core Transcriptional and Functional Signatures in Lymphoid and Mucosal Sites. Cell Rep 20, 2921–2934 (2017). 28930685 10.1016/j.celrep.2017.08.078 PMC5646692

[27] Godec J. Compendium of Immune Signatures Identifies Conserved and Species-Specific Biology in Response to Inflammation. Immunity 44, 194–206 (2016). 26795250 10.1016/j.immuni.2015.12.006 PMC5330663

[28] Yamaguchi U. Distinct gene expression-defined classes of gastrointestinal stromal tumor. J Clin Oncol 26, 4100–8 (2008). 18757323 10.1200/JCO.2007.14.2331

[29] Yu X. Pan-cancer gammadelta TCR analysis uncovers clonotype diversity and prognostic potential. Cell Rep Med 5, 101764 (2024). 10.1016/j.xcrm.2024.101764 PMC11513832 39368482

[30] Vitiello G.A. Differential immune profiles distinguish the mutational subtypes of gastrointestinal stromal tumor. J Clin Invest 129, 1863–1877 (2019). 30762585 10.1172/JCI124108 PMC6486334

[31] Gold J.S. Development and validation of a prognostic nomogram for recurrence-free survival after complete surgical resection of localised primary gastrointestinal stromal tumour: a retrospective analysis. Lancet Oncol 10, 1045–52 (2009). 19793678 10.1016/S1470-2045(09)70242-6 PMC3175638

[32] Zhou J., Kang N., Cui L., Ba D. & He W. Anti-gammadelta TCR antibody-expanded gammadelta T cells: a better choice for the adoptive immunotherapy of lymphoid malignancies. Cell Mol Immunol 9, 34–44 (2012). 21666706 10.1038/cmi.2011.16 PMC4002925

[33] Lopes N. Distinct metabolic programs established in the thymus control effector functions of gammadelta T cell subsets in tumor microenvironments. Nat Immunol 22, 179–192 (2021). 33462452 10.1038/s41590-020-00848-3 PMC7610600

[34] Lien S.C. Tumor reactive gammadelta T cells contribute to a complete response to PD-1 blockade in a Merkel cell carcinoma patient. Nat Commun 15, 1094 (2024). 38321065 10.1038/s41467-024-45449-y PMC10848161

[35] Seifert A.M. PD-1/PD-L1 Blockade Enhances T-cell Activity and Antitumor Efficacy of Imatinib in Gastrointestinal Stromal Tumors. Clin Cancer Res 23, 454–465 (2017). 27470968 10.1158/1078-0432.CCR-16-1163 PMC5241182

[36] Ou L. Dichotomous and stable gamma delta T-cell number and function in healthy individuals. J Immunother Cancer 9(2021). 10.1136/jitc-2020-002274 PMC8137237 34011536

[37] Harly C. Human gammadelta T cell sensing of AMPK-dependent metabolic tumor reprogramming through TCR recognition of EphA2. Sci Immunol 6(2021). 10.1126/sciimmunol.aba9010 34330813

[38] Groh V. Broad tumor-associated expression and recognition by tumor-derived gamma delta T cells of MICA and MICB. Proc Natl Acad Sci U S A 96, 6879–84 (1999). 10359807 10.1073/pnas.96.12.6879 PMC22010

[39] Yuan L. Phosphoantigens glue butyrophilin 3A1 and 2A1 to activate Vgamma9Vdelta2 T cells. Nature 621, 840–848 (2023). 37674084 10.1038/s41586-023-06525-3 PMC10533412

[40] Cavnar M.J. KIT oncogene inhibition drives intratumoral macrophage M2 polarization. J Exp Med 210, 2873–86 (2013). 24323358 10.1084/jem.20130875 PMC3865475

[41] Medina B.D. Oncogenic kinase inhibition limits Batf3-dependent dendritic cell development and antitumor immunity. J Exp Med 216, 1359–1376 (2019). 31000683 10.1084/jem.20180660 PMC6547861

[42] Ferry G.M. A Simple and Robust Single-Step Method for CAR-Vdelta1 gammadeltaT Cell Expansion and Transduction for Cancer Immunotherapy. Front Immunol 13, 863155 (2022). 10.3389/fimmu.2022.863155 PMC9197253 35711450

[43] Makkouk A. Off-the-shelf Vdelta1 gamma delta T cells engineered with glypican-3 (GPC-3)-specific chimeric antigen receptor (CAR) and soluble IL-15 display robust antitumor efficacy against hepatocellular carcinoma. J Immunother Cancer 9(2021). 10.1136/jitc-2021-003441 PMC8679077 34916256

[44] Sterner R.C. & Sterner R.M. CAR-T cell therapy: current limitations and potential strategies. Blood Cancer J 11, 69 (2021). 33824268 10.1038/s41408-021-00459-7 PMC8024391

[45] Hao Y. Dictionary learning for integrative, multimodal and scalable single-cell analysis. Nat Biotechnol 42, 293–304 (2024). 37231261 10.1038/s41587-023-01767-y PMC10928517

[46] Kanehisa M., Furumichi M., Tanabe M., Sato Y. & Morishima K. KEGG: new perspectives on genomes, pathways, diseases and drugs. Nucleic Acids Res 45, D353–D361 (2017). 27899662 10.1093/nar/gkw1092 PMC5210567

[47] Aibar S. SCENIC: single-cell regulatory network inference and clustering. Nat Methods 14, 1083–1086 (2017). 28991892 10.1038/nmeth.4463 PMC5937676

[48] Van de Sande B. A scalable SCENIC workflow for single-cell gene regulatory network analysis. Nat Protoc 15, 2247–2276 (2020). 32561888 10.1038/s41596-020-0336-2

[49] Dobin A. STAR: ultrafast universal RNA-seq aligner. Bioinformatics 29, 15–21 (2013). 23104886 10.1093/bioinformatics/bts635 PMC3530905

[50] Liao Y., Smyth G.K. & Shi W. featureCounts: an efficient general purpose program for assigning sequence reads to genomic features. Bioinformatics 30, 923–30 (2014). 24227677 10.1093/bioinformatics/btt656

[51] Korotkevich G. Fast gene set enrichment analysis. bioRxiv, 060012 (2021).

[52] Liberzon A. The Molecular Signatures Database (MSigDB) hallmark gene set collection. Cell Syst 1, 417–425 (2015). 26771021 10.1016/j.cels.2015.12.004 PMC4707969

[53] Newman A.M. Determining cell type abundance and expression from bulk tissues with digital cytometry. Nat Biotechnol 37, 773–782 (2019). 31061481 10.1038/s41587-019-0114-2 PMC6610714

